# Cytopenias With HIV in the Era of Highly Active Antiretroviral Therapy: Are We Winning the Battle?

**DOI:** 10.1002/hsr2.72065

**Published:** 2026-03-15

**Authors:** Emmanuel Ifeanyi Obeagu

**Affiliations:** ^1^ Department of Biomedical and Laboratory Science Africa University Mutare Zimbabwe

**Keywords:** bone marrow suppression, cytopenias, HAART, hematological complications, HIV

## Abstract

**Background and Aim:**

Cytopenias remain among the most frequent hematologic complications of HIV infection, affecting treatment outcomes and quality of life. The introduction of highly active antiretroviral therapy has markedly reduced their incidence by restoring immune function and suppressing viral replication. However, the magnitude of this progress varies widely between developed and underdeveloped regions. While cytopenias have declined substantially in developed countries due to early HAART initiation and advanced monitoring, they remain prevalent in low‐resource settings where delayed diagnosis, coinfections, and limited healthcare infrastructure persist. This review aims to synthesize current evidence on HIV‐associated cytopenias in the HAART era, comparing regional trends, underlying mechanisms, and management outcomes.

**Methods:**

A comprehensive literature search was conducted across PubMed, Scopus, Web of Science, and Google Scholar for studies published from 2000 to 2025. Search terms included *HIV, cytopenia, anemia, thrombocytopenia, leukopenia*, and *HAART*. Eligible peer‐reviewed articles, reviews, and reports addressing the epidemiology, pathogenesis, and clinical management of cytopenias in adults living with HIV were included.

**Results:**

Evidence shows a substantial decline in the prevalence and severity of cytopenias in developed regions, largely due to early HAART initiation, improved monitoring, and access to less myelotoxic drug regimens. In contrast, in underdeveloped regions, cytopenias remain common, driven by delayed diagnosis, limited HAART coverage, malnutrition, coinfections, and inadequate laboratory capacity. Persistent anemia and thrombocytopenia continue to undermine quality of life and treatment outcomes in these settings.

**Conclusion:**

The global fight against HIV‐associated cytopenias illustrates both the success and inequity of the HAART era. While developed countries have largely overcome the burden, underdeveloped regions continue to struggle with preventable hematologic complications. To fully “win the battle,” there is an urgent need for equitable access to early diagnosis, optimized therapy, and comprehensive hematologic support worldwide.

## 1 Introduction

Cytopenias—defined as a reduction in one or more major blood cell lines (erythrocytes, leukocytes, or platelets)—remain a significant hematologic complication of human immunodeficiency virus (HIV) infection. Since the emergence of the acquired immunodeficiency syndrome (AIDS) epidemic, hematologic abnormalities have been recognized as both diagnostic hallmarks and prognostic indicators of disease progression [1, 2]. In the pre–highly active antiretroviral therapy (HAART) era, cytopenias were common and often severe, reflecting the profound bone marrow suppression and immune dysregulation caused by uncontrolled viral replication, opportunistic infections, and nutritional deficiencies. The introduction of HAART has dramatically altered the natural history of HIV infection, transforming it from a fatal disease to a chronic, manageable condition. However, despite this therapeutic revolution, cytopenias continue to pose clinical and public health challenges, particularly in resource‐limited settings [3, 4, 5].

The burden of cytopenias among people living with HIV (PLWH) varies markedly between developed and underdeveloped regions. In high‐income countries, access to early diagnosis, optimized HAART regimens, and improved supportive care have substantially reduced the prevalence and severity of hematologic complications [6, 7, 8]. Conversely, in low‐ and middle‐income countries (LMICs), where HIV remains highly prevalent, cytopenias persist as a major cause of morbidity and mortality. Multiple factors contribute to this disparity: delayed presentation, limited access to HAART, coinfections such as malaria and tuberculosis, malnutrition, and inadequate laboratory infrastructure for diagnosis and monitoring. These challenges are compounded by socioeconomic barriers and health system limitations that impede comprehensive management of hematologic disorders in HIV‐infected individuals [9, 10, 11, 12]. Cytopenias in the context of HIV infection are multifactorial in origin. They may result from direct viral invasion of bone marrow progenitor cells, immune‐mediated destruction, chronic inflammation, or drug‐induced myelosuppression. Anemia remains the most frequent manifestation, often associated with fatigue, poor quality of life, and reduced survival. Thrombocytopenia may predispose to bleeding complications, while leukopenia increases susceptibility to infections. Understanding the complex interplay between HIV pathogenesis, host immune response, and therapeutic interventions is therefore critical to improving clinical outcomes [13, 14, 15].

The advent of HAART has significantly reduced HIV‐associated cytopenias through suppression of viral replication and partial restoration of hematopoiesis. Nonetheless, the persistence or emergence of cytopenias despite therapy—referred to as “HAART‐era cytopenias”—raises new clinical questions. Are these residual abnormalities indicative of incomplete immune recovery, drug toxicity, or the impact of comorbid conditions? Moreover, how do these patterns differ between populations with robust healthcare systems and those in resource‐limited environments? Addressing these questions is vital for evaluating whether global HIV treatment efforts are truly winning the battle against hematologic complications [16, 17, 18]. The present review aims to synthesize current evidence on cytopenias in the era of HAART, highlighting both the global progress achieved and the persisting inequities that hinder universal success. Special attention is given to the experiences of low‐resource settings, where the fight against HIV remains intertwined with broader challenges of poverty, limited access to healthcare, and inadequate laboratory support. By comparing trends between developed and underdeveloped regions, this review seeks to provide a balanced perspective on the evolving landscape of HIV‐associated cytopenias and to identify strategies for ensuring that hematologic recovery becomes a universal reality for all individuals living with HIV.

## 2 Aim

The aim of this review is to provide a comprehensive analysis of cytopenias in HIV‐infected individuals, examining the various types, underlying mechanisms, and clinical implications of these hematologic abnormalities in the context of HIV disease progression and treatment.

## 3 Methods

A narrative review was conducted using PubMed, Scopus, Web of Science, and Google Scholar for studies published between 2000 and 2025. Search terms included *HIV, cytopenia, anemia, leukopenia, thrombocytopenia*, and *HAART*. Peer‐reviewed articles, systematic reviews, and reports examining the prevalence, pathophysiology, and management of cytopenias in adults living with HIV were included. Data were extracted to highlight regional variations between developed and underdeveloped settings, focusing on the effects of HAART and associated clinical outcomes. The Scale for the Assessment of Narrative Review Articles (SANRA) guided quality evaluation and synthesis.

### 3.1 Types and Mechanisms of Cytopenias in HIV

Cytopenias are common hematological abnormalities in individuals with HIV and are typically classified into three main types: anemia, leukopenia (particularly neutropenia), and thrombocytopenia. These may occur in isolation or as combined cytopenias such as pancytopenia. Each type presents with distinct clinical implications and arises from a complex interplay of viral, host, and treatment‐related factors [2, 19].

#### 3.1.1 Anemia

Anemia is the most frequently observed cytopenia in HIV patients, with multiple underlying mechanisms. Chronic inflammation associated with HIV leads to elevated levels of inflammatory cytokines such as interleukin‐1, tumor necrosis factor‐alpha (TNF‐α), and interferon‐gamma, which suppress erythropoietin production and impair erythroid progenitor cell function. Direct HIV infection of bone marrow stromal cells and altered hematopoietic microenvironments contribute to ineffective erythropoiesis. Moreover, coinfections (e.g., parvovirus B19, tuberculosis), nutritional deficiencies (iron, vitamin B12, folate), and gastrointestinal malabsorption further exacerbate anemia. Drug‐induced myelosuppression, particularly from zidovudine, is also a well‐recognized cause.

#### 3.1.2 Leukopenia/Neutropenia

Leukopenia, especially neutropenia, can result from direct viral suppression of granulocyte precursors or immune‐mediated destruction of circulating leukocytes. HIV can infect CD34+ hematopoietic progenitor cells and impair granulocyte colony‐stimulating factor (G‐CSF) signaling, leading to reduced neutrophil production. Additionally, antiretroviral drugs and antimicrobials like ganciclovir, cotrimoxazole, and chemotherapy agents contribute to neutropenia. Neutropenia predisposes patients to opportunistic infections, especially in advanced disease stages or among those with concomitant bone marrow suppression from malignancies or other chronic infections [20, 21].

#### 3.1.3 Thrombocytopenia

Thrombocytopenia occurs in both early and late HIV disease and may be due to immune‐mediated platelet destruction or impaired platelet production. Immune thrombocytopenic purpura (ITP) is a common manifestation, resulting from the production of anti‐platelet antibodies that accelerate peripheral destruction. Additionally, HIV can infect megakaryocytes, impairing their ability to produce platelets. Coinfections such as hepatitis C and medications like interferon‐alpha may worsen thrombocytopenia. Although HAART has been shown to improve platelet counts in many patients, persistent thrombocytopenia still occurs and is often multifactorial [21, 22, 23].

### 3.2 Impact of HAART on Cytopenias

The introduction of HAART in the late 1990s revolutionized the management of HIV infection, leading to significant reductions in viral load, enhanced immune recovery, and improved survival rates. Despite these successes, cytopenias remain a persistent challenge in the care of HIV‐infected individuals.

#### 3.2.1 Reduction in Cytopenia Prevalence

HAART has led to substantial improvements in hematological profiles, particularly through effective viral suppression and immune reconstitution. With the restoration of CD4+ T‐cell counts and the reduction in viral replication, patients on HAART generally experience fewer complications related to cytopenias. Anemia, neutropenia, and thrombocytopenia are less frequent in patients with optimal adherence to ART, and the severity of these conditions tends to decrease after the initiation of therapy. Studies have shown that anemia rates, for instance, significantly improve in individuals who achieve sustained viral suppression with modern regimens. Similarly, neutrophil and platelet counts tend to stabilize in patients whose HIV is well‐controlled, reducing the risks of infections and bleeding [24, 25].

#### 3.2.2 Persistent Cytopenias Despite Viral Suppression

Although HAART has reduced the incidence of cytopenias, these hematological abnormalities persist in certain patients, particularly in those with suboptimal viral control or advanced immunosuppression. For example, even with effective viral suppression, some individuals continue to experience moderate to severe anemia or thrombocytopenia. This is partly due to the long‐term effects of chronic inflammation and immune activation associated with HIV. Despite viral suppression, residual immune dysfunction, including the increased release of pro‐inflammatory cytokines like TNF‐α, may inhibit hematopoiesis, leading to sustained cytopenias. Additionally, many patients receiving HAART, especially those on older nucleoside reverse transcriptase inhibitors (NRTIs) such as zidovudine, still face drug‐induced bone marrow suppression [26, 27, 28].

#### 3.2.3 Drug‐Related Myelosuppression and Adverse Effects

While HAART has improved outcomes for most patients, certain antiretroviral medications can exacerbate cytopenias. Older NRTIs, particularly zidovudine, have been associated with dose‐dependent myelosuppression, leading to anemia and neutropenia. Although newer ART regimens with fewer side effects (such as integrase inhibitors and non‐NRTIs) are now available, they are not without potential toxicities. Furthermore, certain co‐treatment drugs, such as those used to manage opportunistic infections (e.g., cotrimoxazole, ganciclovir), can also cause myelosuppression. In cases of drug‐related cytopenias, switching to alternative antiretroviral regimens or adjusting co‐treatment strategies is often necessary to mitigate hematological side effects [29, 30, 31, 32].

#### 3.2.4 Co‐Infections and Comorbidities Contributing to Cytopenias

Another key factor in the persistence of cytopenias, even in the era of HAART, is the ongoing presence of co‐infections and comorbidities. HIV‐infected individuals are at increased risk for co‐infections such as tuberculosis, malaria, and viral hepatitis, all of which can contribute to hematological abnormalities. For instance, malaria‐induced hemolysis can exacerbate anemia, while tuberculosis and hepatitis C may worsen thrombocytopenia. In resource‐limited settings, where the burden of these infections is higher, cytopenias may persist despite ART, complicating clinical management. Furthermore, chronic kidney disease, which is a known complication of HIV and its treatment, can also contribute to anemia through impaired erythropoiesis [33, 34, 35, 36].

#### 3.2.5 The Role of HAART in Long‐Term Hematologic Recovery

While HAART has significantly improved hematologic outcomes, full recovery from cytopenias may not always occur, particularly in patients with long‐standing HIV infection or advanced immunosuppression. The degree of bone marrow recovery depends on several factors, including the timing of ART initiation, CD4+ T‐cell count, and the extent of viral suppression. Early initiation of HAART before the onset of severe immunosuppression is associated with better recovery of hematologic function, emphasizing the importance of early diagnosis and treatment. However, in individuals with established cytopenias prior to starting HAART, full hematologic normalization may not always be achieved, and ongoing monitoring and management are necessary [37, 38, 39, 40].

### 3.3 Clinical Implications and Management of Cytopenias in HIV

#### 3.3.1 Clinical Implications of Cytopenias in HIV

Cytopenias in HIV‐infected individuals can have significant clinical implications, impacting both the overall management of the disease and the patient's quality of life. The presence of anemia, neutropenia, or thrombocytopenia may indicate disease progression, immune suppression, or the development of complications such as opportunistic infections or malignancies. Anemia, for instance, has been linked to decreased survival and poorer clinical outcomes in HIV patients, particularly those with advanced immunosuppression. Neutropenia increases susceptibility to bacterial and fungal infections, while thrombocytopenia poses risks for bleeding and requires careful management to prevent life‐threatening hemorrhages. In the context of HIV treatment, persistent cytopenias can complicate the choice of antiretroviral therapy (ART). For example, some antiretroviral drugs, especially older NRTIs like zidovudine, are known to cause myelosuppression, exacerbating existing cytopenias. Additionally, patients with thrombocytopenia or neutropenia may need dose adjustments or substitutions in their ART regimens to avoid further hematologic toxicity. For clinicians, managing cytopenias in HIV‐infected patients requires a comprehensive approach, considering not only the direct effects of HIV and ART but also the influence of comorbidities, coinfections, and the patient's overall immune status. Moreover, cytopenias often correlate with higher healthcare utilization, increased hospitalizations, and extended durations of illness due to opportunistic infections or other complications. This adds a layer of complexity to managing HIV, especially in resource‐limited settings where timely interventions for hematologic abnormalities may not be as readily available. Therefore, early detection, vigilant monitoring, and appropriate therapeutic interventions are critical to improving the clinical outcomes for HIV patients with cytopenias [41, 42].

#### 3.3.2 Management Strategies for Cytopenias in HIV

Managing cytopenias in HIV requires a multi‐faceted approach tailored to the individual patient's needs, the type of cytopenia, and the underlying causes. The management of anemia, for instance, primarily focuses on correcting underlying nutritional deficiencies (e.g., iron, folate, or vitamin B12), treating co‐infections, and, when appropriate, adjusting ART regimens to minimize drug‐induced myelosuppression. In some cases, erythropoiesis‐stimulating agents (ESAs) may be prescribed to stimulate red blood cell production in individuals with significant anemia, although their use should be carefully monitored due to potential side effects. In cases of neutropenia, the primary goal is to reduce the risk of infections while stimulating neutrophil production. G‐CSF can be used to boost neutrophil levels in individuals with severe neutropenia. However, G‐CSF should be used cautiously, especially in patients with a history of certain cancers, as it can potentially stimulate tumor growth. Additionally, prophylactic antimicrobials (such as cotrimoxazole) are often prescribed to prevent bacterial infections in neutropenic patients. In patients who are on drugs known to induce neutropenia, such as ganciclovir, dose adjustments or discontinuation of the offending agent may be necessary. For thrombocytopenia, treatment often involves addressing any underlying causes, such as ITP, where corticosteroids or intravenous immunoglobulin (IVIG) may be used to suppress platelet destruction. If the thrombocytopenia is related to HIV itself, platelet transfusions may be required for patients with severe thrombocytopenia or active bleeding. The management of thrombocytopenia in HIV patients also includes careful monitoring of bleeding risks, as platelet dysfunction may also occur in advanced HIV infection, even in the absence of significant thrombocytopenia [43, 44].

#### 3.3.3 Management of Cytopenias in the Context of HAART

One of the key challenges in managing cytopenias in HIV patients receiving HAART is balancing the need for effective viral suppression with the risk of hematologic toxicity. For patients who develop drug‐induced cytopenias, switching to an alternative ART regimen may be required. For example, if zidovudine‐induced anemia is suspected, switching to a regimen containing tenofovir or other NRTIs with a lower incidence of myelosuppression may be appropriate. It is important to note that newer ART regimens, such as those based on integrase inhibitors (e.g., dolutegravir or raltegravir), have shown better hematological safety profiles compared to older regimens. The timing of ART initiation also plays a critical role in preventing or managing cytopenias. Early initiation of HAART, particularly in individuals with higher CD4+ T‐cell counts, is associated with improved immune recovery and a lower incidence of cytopenias. In contrast, delayed initiation in individuals with advanced HIV may result in prolonged periods of immune suppression, further increasing the risk of persistent cytopenias and related complications. In the case of co‐infections or comorbidities, careful coordination between specialists is essential. For example, if a patient has both HIV and tuberculosis, the combination of ART with anti‐tuberculosis drugs may require adjustments to minimize interactions and reduce the risk of drug‐induced cytopenias. Similarly, patients with HIV and hepatitis C, who are often treated with interferon‐based regimens, may require dose adjustments or close monitoring of blood counts due to the risk of both drug‐induced and HIV‐related cytopenias [45, 46, 47].

#### 3.3.4 Preventive Strategies and Monitoring

Prevention of cytopenias in HIV involves proactive screening and early intervention strategies. Routine blood tests to monitor hemoglobin levels, white blood cell counts, and platelet counts should be an integral part of HIV care, especially for patients starting or switching ART. Regular monitoring can help identify cytopenias early, allowing for timely intervention before more severe complications arise. Preventive strategies may also include prophylactic treatments for opportunistic infections and vaccinations, particularly in patients with low CD4+ T‐cell counts who are at higher risk for infections that can exacerbate cytopenias. Furthermore, nutritional support is essential for maintaining optimal hematologic health. Ensuring adequate intake of iron, folate, vitamin B12, and other micronutrients can prevent deficiencies that may contribute to anemia. In resource‐limited settings, where nutritional deficiencies are more prevalent, the importance of supplementation cannot be overstated. In some cases, addressing parasitic infections like malaria, which is a common co‐infection in HIV patients, may significantly improve hematologic parameters [48, 49].

### 3.4 Persistent Challenges in Low‐Resource Settings

#### 3.4.1 Limited Access to Diagnostics and Monitoring

One of the most significant challenges in managing cytopenias in HIV‐infected individuals in low‐resource settings is limited access to essential diagnostic tools and monitoring services. Routine blood tests, such as complete blood counts (CBC), are critical for detecting cytopenias early and guiding appropriate management. However, in many low‐resource settings, access to these diagnostic services is often limited, particularly in rural or underserved areas. Without timely access to these tests, it becomes difficult for healthcare providers to detect cytopenias before they lead to severe complications, such as infections or bleeding events. This lack of monitoring further complicates the effective management of HIV patients, as adjustments to ART or other treatments may be delayed. Moreover, the availability of specialized diagnostic tests, such as bone marrow aspirates or viral load monitoring, may be scarce. In the absence of comprehensive monitoring, clinicians may not be able to assess the severity of cytopenias or determine their exact etiology, which can result in suboptimal care. For instance, distinguishing between cytopenias caused by HIV itself, ART‐induced toxicity, or co‐infections requires a comprehensive approach, which is often difficult in resource‐limited settings due to the lack of diagnostic infrastructure [50, 51].

#### 3.4.2 Inadequate Access to Medications and ART

Another persistent challenge is the limited availability of first‐line and second‐line ART in low‐resource settings. While HAART has significantly improved survival and immune recovery in HIV patients, many individuals in resource‐constrained environments still do not have access to the most effective ART regimens. This is particularly true for second‐line and third‐line regimens that are used when patients fail to respond to first‐line treatments or experience intolerable side effects such as myelosuppression. The unavailability of newer ART agents with better hematologic safety profiles means that many patients continue to receive older regimens, which may exacerbate cytopenias. Additionally, the availability of supportive medications, such as ESAs, G‐CSF, and IVIG for managing cytopenias, is often limited in low‐resource settings. These therapies can be crucial for addressing severe cytopenias and preventing complications. However, their high cost and logistical challenges, such as the need for refrigeration or specialized administration, often make them inaccessible to many patients [52, 53, 54].

#### 3.4.3 Co‐Infections and Comorbidities in Resource‐Limited Settings

In low‐resource settings, HIV patients are often burdened with multiple co‐infections, such as tuberculosis, malaria, and hepatitis, which can exacerbate cytopenias and complicate treatment. For instance, the high prevalence of tuberculosis in many sub‐Saharan African countries often requires co‐treatment with antiretroviral drugs and anti‐tuberculosis medications, which may interact and increase the risk of hematologic side effects. Similarly, malaria‐induced hemolysis can worsen anemia, particularly in individuals with preexisting HIV‐related cytopenias. Managing these co‐infections effectively requires a coordinated approach, which can be difficult to implement in settings with limited healthcare resources. Inadequate access to medications for co‐infections and the lack of specialized care to monitor for drug interactions and side effects can lead to poorer outcomes for patients. In addition, the burden of parasitic infections like malaria can further stress the already compromised immune system of HIV patients, making it harder to manage cytopenias [55, 56, 57].

#### 3.4.4 Health Workforce Limitations and Training Needs

The shortage of trained healthcare workers is another significant challenge in low‐resource settings, particularly in rural or isolated areas. The management of HIV‐related cytopenias requires skilled healthcare professionals who can accurately diagnose and treat these conditions, as well as the capacity to adjust ART regimens and provide supportive care. In many low‐resource settings, however, there is a critical shortage of trained clinicians and laboratory technicians, which hampers the ability to provide quality care. Additionally, many healthcare workers may not be fully trained to recognize and manage the specific hematologic complications that can arise in HIV‐infected individuals. Continuous professional development and training programs are often inadequate, leading to gaps in knowledge regarding the latest advances in HIV treatment, management of cytopenias, and co‐infection management. This lack of expertise can result in delayed or inappropriate treatment, which may worsen outcomes for HIV patients with cytopenias [58, 59, 60].

#### 3.4.5 Financial Constraints and Healthcare Infrastructure

Financial constraints are a major factor contributing to the challenges in managing cytopenias in HIV patients in low‐resource settings. Governments and healthcare systems in many LMICs face limited budgets for healthcare, which restricts the availability of medications, diagnostic tools, and treatment options for HIV patients. As a result, the quality of care for patients with HIV‐related cytopenias can be compromised, and access to life‐saving interventions, such as blood transfusions, G‐CSFs, and blood monitoring, can be limited. In addition, the lack of healthcare infrastructure—such as the absence of fully equipped clinics or hospitals, inadequate storage facilities for medications, and unreliable transportation for drug distribution—further complicates the management of cytopenias. Without a robust healthcare system that can provide timely and comprehensive care, patients in low‐resource settings remain vulnerable to complications arising from cytopenias, even in the era of HAART [61, 62, 63].

### 3.5 Cytopenias in Developed Regions: A Declining Burden

In developed regions, the landscape of HIV‐associated cytopenias has changed profoundly with the advent and widespread accessibility of HAART. Early initiation of therapy, guided by robust diagnostic systems and well‐resourced healthcare infrastructures, has resulted in a marked decline in the prevalence and severity of cytopenias among PLWH. Routine laboratory monitoring, including CBCs and bone marrow assessments when indicated, enables timely detection and management of hematologic abnormalities before they progress to clinical complications [59]. The availability of potent and less myelotoxic antiretroviral agents has further minimized treatment‐related cytopenias. Contemporary HAART regimens—particularly integrase inhibitor‐based combinations—have demonstrated superior hematologic tolerability compared with earlier NRTIs such as zidovudine, which were historically linked to anemia and neutropenia. Furthermore, access to adjunctive therapies such as erythropoietin‐stimulating agents, iron supplementation, and hematopoietic growth factors has improved patient outcomes and enhanced quality of life [60].

Advanced monitoring tools in developed regions also allow clinicians to distinguish between HAART‐related cytopenias and those arising from opportunistic infections, autoimmune processes, or bone marrow suppression due to malignancy. This precision in diagnosis supports individualized management strategies and contributes to sustained hematologic stability. Moreover, multidisciplinary HIV care models—integrating infectious disease specialists, hematologists, nutritionists, and social support services—play a crucial role in maintaining comprehensive patient well‐being [61, 62]. The success seen in developed regions underscores the transformative potential of early HAART initiation, consistent follow‐up, and access to advanced medical technologies. Cytopenias, once a hallmark of HIV progression, have now become uncommon and generally reversible in these settings. The experience of developed countries serves as a benchmark and a source of valuable lessons for improving hematologic outcomes in resource‐limited environments (Table 1) [63].

**Table 1:** Cytopenias in developed regions.

Aspect	Key features and findings	Implications
Prevalence and trends	Significant decline in anemia, leukopenia, and thrombocytopenia since the introduction of HAART. Cytopenias are now infrequent and generally mild.	Reflects successful viral suppression and immune restoration through early HAART initiation.
Early HAART initiation	Prompt therapy reduces bone marrow suppression and prevents advanced immune depletion.	Early treatment initiation is critical for hematologic recovery and improved survival.
Drug regimen improvements	Transition from zidovudine‐based to integrase inhibitor–based regimens has reduced myelotoxicity.	Modern HAART combinations minimize hematologic toxicity and improve adherence.
Advanced monitoring systems	Routine full blood counts, hematologic profiling, and access to bone marrow diagnostics are standard.	Enables early detection, precise management, and prevention of severe cytopenias.
Supportive care	Access to nutritional supplementation, erythropoietin, and hematopoietic growth factors enhances outcomes.	Comprehensive care prevents chronic cytopenias and improves quality of life.
Research and surveillance	Continuous research and data integration guide therapeutic adjustments and long‐term safety monitoring.	Sustains evidence‐based improvements in HIV hematologic care.

### 3.6 Are We Winning the Battle? Cytopenias in the Era of HAART

The introduction of HAART has undoubtedly revolutionized the management of HIV, leading to significant reductions in morbidity, mortality, and the incidence of opportunistic infections. For HIV patients, HAART represents a beacon of hope, offering long‐term viral suppression and improved immune function. However, while HAART has vastly improved survival rates and life quality, it has not been a panacea for all HIV‐related complications. Among these, cytopenias—conditions characterized by a reduction in the number of blood cells—remain a persistent challenge. The question arises: *Are we winning the battle* against cytopenias in HIV‐infected individuals? Cytopenias, which can affect red blood cells, white blood cells, or platelets, are a common complication in HIV‐infected individuals, presenting as anemia, neutropenia, or thrombocytopenia. The mechanisms behind these hematologic abnormalities are multifactorial, involving both the direct effects of the HIV virus on hematopoiesis and the side effects of ART. While some cytopenias in HIV can be attributed to opportunistic infections, nutritional deficiencies, and chronic inflammation, ART itself, although life‐saving, can also contribute to hematologic abnormalities, complicating patient management. Despite HAART's success in controlling the virus, cytopenias often continue to impede patient health, triggering the question of whether progress in managing these complications matches the advancements in HIV treatment [64].

The benefits of HAART in suppressing viral replication and restoring immune function are undisputed. However, as the number of patients living longer with HIV increases, the long‐term side effects of ART, particularly cytopenias, are becoming more apparent. For example, certain antiretroviral drugs, such as zidovudine (an NRTI), are known to induce bone marrow suppression, leading to anemia and neutropenia. These hematologic issues can further compromise an already fragile immune system, rendering patients more susceptible to infections and increasing the complexity of their care. Thus, while HAART is effective in managing HIV, it also raises new challenges related to hematologic side effects, presenting a dilemma for healthcare providers. As the global community continues to push for the elimination of HIV as a public health threat, addressing the question of whether we are “winning the battle” against cytopenias becomes essential. In high‐resource settings, advances in ART have led to improved management strategies and the development of newer, more tolerable medications with fewer hematologic side effects. The advent of integrase inhibitors, for example, has provided an alternative to older drugs associated with significant cytopenic risks. However, these advancements are not universally accessible, especially in low‐resource settings where HIV remains a significant health burden. The lack of access to newer ART formulations, diagnostics, and supportive care options means that many HIV‐infected individuals in these regions continue to suffer from untreated or inadequately managed cytopenias [65].

Low‐resource settings face unique challenges that complicate the effective management of cytopenias. Limited access to ART, diagnostic tools, and healthcare infrastructure exacerbates the prevalence of cytopenias, often resulting in poor patient outcomes. Routine blood testing to monitor hematologic health is frequently unavailable, and the high cost of newer, more effective ART regimens means that many patients are still receiving older, more toxic therapies. The lack of trained healthcare providers and treatment adherence issues further exacerbate the difficulty of managing cytopenias in these regions. As a result, the battle against these hematologic complications remains uphill in many parts of the world, and this disparity in care must be addressed if we are to truly declare victory over HIV and its associated complications [64]. The emergence of cytopenias in the context of HIV necessitates a nuanced approach to patient management, balancing the benefits of ART with the risk of hematologic toxicity. Regular monitoring of blood counts, timely adjustments to treatment regimens, and supportive therapies such as ESAs or G‐CSFs are essential for managing these complications in HIV patients. However, in resource‐limited settings, where these interventions may not be available, clinicians must rely on a combination of basic care principles, early recognition, and creative solutions to manage cytopenias effectively (Table 2) [65].

**Table 2:** Are we winning the battle? Cytopenias in the era of HAART.

Domain	Pre‐HAART era	HAART era	Current challenges	Overall assessment
Prevalence of cytopenias	Extremely high rates of anemia, leukopenia, and thrombocytopenia due to uncontrolled viral replication and opportunistic infections.	Marked reduction in prevalence, particularly in regions with early and sustained HAART coverage.	Persistent cytopenias in underdeveloped regions due to delayed treatment and comorbidities.	Significant improvement, but uneven global progress.
Pathophysiology	Dominated by direct HIV bone marrow suppression and immune dysregulation.	Improved marrow recovery due to viral control and immune reconstitution.	Drug‐induced myelosuppression and chronic inflammation remain in some patients.	Partial restoration of normal hematopoiesis.
Therapeutic landscape	Limited or no access to antiretroviral therapy; reliance on supportive treatment.	Widespread HAART availability with optimized, less toxic regimens.	Inconsistent drug access and monitoring in resource‐limited settings.	Winning in access, but lagging in equity.
Monitoring and diagnostics	Minimal hematologic monitoring and poor laboratory capacity.	Regular blood monitoring standard in developed regions.	Weak laboratory infrastructure in many low‐income countries.	Strong in high‐income regions, fragile in low‐resource settings.
Patient outcomes	High morbidity and mortality related to cytopenias.	Improved survival, reduced hospitalizations, and enhanced quality of life.	Persistent anemia and thrombocytopenia undermine outcomes in low‐resource contexts.	Substantial survival gains, though not universal.
Global outlook	HIV‐associated cytopenias a major health burden.	HAART has transformed HIV into a manageable chronic condition.	Equity gaps and health system limitations still exist.	We are winning the battle—but not yet the war.

## 4 Conclusion

Cytopenias remain an important clinical concern in HIV management despite the transformative success of HAART. The widespread availability of HAART has substantially reduced the incidence and severity of hematologic complications in developed countries, underscoring the therapeutic progress achieved through early diagnosis, consistent treatment, and comprehensive patient monitoring. However, in underdeveloped and resource‐limited settings, cytopenias continue to impose a significant burden, often reflecting systemic barriers such as delayed treatment initiation, coinfections, malnutrition, and inadequate laboratory capacity. The persistence of cytopenias in the HAART era highlights that biomedical progress alone is insufficient without equitable access and sustained health system strengthening. Bridging the gap between regions requires deliberate policy actions, capacity building, and equitable distribution of diagnostic and therapeutic resources. Addressing the disparities in care will ensure that the benefits of HAART extend beyond viral suppression to full hematologic restoration and improved quality of life for all PLWH.

## Author Contributions

**Emmanuel Ifeanyi Obeagu:** conceptualization, writing – original draft, methodology, validation, visualization, writing – review and editing, supervision, resources.

## Funding

The author received no specific funding for this work.

## Conflicts of Interest

The author declares no conflicts of interest.

## Transparency Statement

The lead author Emmanuel Ifeanyi Obeagu affirms that this manuscript is an honest, accurate, and transparent account of the study being reported; that no important aspects of the study have been omitted; and that any discrepancies from the study as planned (and, if relevant, registered) have been explained.

## Data Availability

The author has nothing to report.
